# Supplementary material for: The Role of Community Pharmacists in the Detection of Clinically Relevant Drug-Related Problems in Chronic Kidney Disease Patients
Source: Pharmacy (Basel). 2020 May 22;8(2):89. doi: 10.3390/pharmacy8020089 (PMC7355920; doi:10.3390/pharmacy8020089)
Supplement: Supplementary file 1 [file pharmacy-08-00089-s001.zip › Table S1.docx]

Table S1: Assessment of the clinical relevance of pharmacist interventions (Tool: *modified* Chedru)

| **Score** | **Significance** |
| --- | --- |
| *-1* | ***Nuisible***  *The PI can lead to adverse outcomes on clinical status, knowledge, satisfaction, patient adherence and/or quality of life of the patient* |
| 0 | **No clinical impact for the patient**  The intervention is present an objective, financial or informational exclusively or was proposed after the event; it is therefore without consequence for the patient. |
| 1 | **Significant impact**  The intervention increases the efficiency and/or safety and/or quality of life of the patient. |
| 2 | **Very significant impact**  The intervention prevents organ dysfunction, it avoids intensive medical surveillance or an irreversible consequence. |
| 3 | **Vital impact**  The intervention avoids a potentially fatal accident |
