# Supplementary material for: The Role of Community Pharmacists in the Detection of Clinically Relevant Drug-Related Problems in Chronic Kidney Disease Patients
Source: Pharmacy (Basel). 2020 May 22;8(2):89. doi: 10.3390/pharmacy8020089 (PMC7355920; doi:10.3390/pharmacy8020089)
Supplement: Supplementary file 1 [file pharmacy-08-00089-s001.zip › Table S2.docx]

| **Drug groups (ATC classification system)** | **n (%)** |
| --- | --- |
| C - Cardiovascular system | 1210 (26.8) |
| Diuretics (C03) | 303 (6.7) |
| Renin-angiotensin system blockers (C09) | 229 (5.1) |
| Hypolipemic drugs (C10) | 196 (4.3) |
| Beta blocking agents (C07) | 185 (4.1) |
| A - Alimentary tract and metabolism | 871 (19.3) |
| Drugs for peptic ulcer and reflux disease (A02 ) | 210 (4.7) |
| Drugs used for diabetes (A10) | 207 (4.6) |
| N – Nervous system | 609 (13.5) |
| Analgesics (N02) | 267 (5.9) |
| Psycholeptics (N05) | 160 (3.5) |
| B – Blood and blood-forming organs | 306 (6.8) |
| Antithrombotics drugs (B01) | 260 (5.8) |
| R- Respiratory system | 262 (5.8) |
| Drugs for obstructive airway diseases (R03) | 150 (3.3) |

Table S2: Most frequently prescribed medicines (n = 4508 prescribed medicines)
