# Supplementary material for: The Role of Community Pharmacists in the Detection of Clinically Relevant Drug-Related Problems in Chronic Kidney Disease Patients
Source: Pharmacy (Basel). 2020 May 22;8(2):89. doi: 10.3390/pharmacy8020089 (PMC7355920; doi:10.3390/pharmacy8020089)
Supplement: Supplementary file 1 [file pharmacy-08-00089-s001.zip › Table S3.docx]

Table S3: Cross-assessment of the clinical relevance of DRPs performed by community and expert pharmacists

|  | **DRP detection by community pharmacists (n (%))** | | | **DRP detection by expert pharmacists (n (%))** | | |
| --- | --- | --- | --- | --- | --- | --- |
|  | Nephrologists | General practitioner 1 | General practitioner 2 | Nephrologists | General practitioner 1 | General practitioner 2 |
| **0** | 39 (49.4) | 38 (46.9) | 32 (41) | 26 (17.8) | 37 (25.2) | 8 (5.4) |
| **1** | 18 (22.8) | 30 (37.0) | 12 (15.4) | 66 (45.2) | 83 (56.5) | 27 (18) |
| **2** | 17 (21.5) | 11 (13.6) | 32 (41.0) | 43 (29.5) | 23 (15.6) | 101 (67.3) |
| **3** | 5 (6.3) | 2 (2.5) | 2 (1.4) | 11 (7.5) | 4 (2.7) | 14 (9.3) |
